# Using the QI Maturity Tool – Modified Ontario Version to assess the state of QI maturity in Ontario’s public health units

**DOI:** 10.1186/s13690-021-00703-3

**Published:** 2021-10-20

**Authors:** Madelyn P. Law, Alex Berry, Nicole Clarke, Graham Hay, Caitlin Muhl, Kelly Pilato, Danielle Hunter, Anna Larson

**Affiliations:** 1 Faculty of Applied Health Sciences, Brock University, St. Catharines, Ontario Canada; 2 Northwestern Health Unit, Kenora, Ontario Canada; 3 Niagara Region Public Health, Thorold, Ontario Canada; 4 North Bay Parry Sound District Health Unit, North Bay, Ontario Canada; 5 Halton Region Health Department, Oakville, Ontario Canada

**Keywords:** Public health, Quality improvement, QI maturity tool

## Abstract

**Background:**

Implementation of quality improvement (QI) practices varies considerably among public health units (PHUs) in Ontario. With the emphasis on continuous quality improvement (CQI) in the revised Ontario Public Health Standards (OPHS), there is a need to understand the level of QI maturity in Ontario’s PHUs. The objective of this research was to establish a baseline understanding of QI maturity in Ontario’s PHUs.

**Methods:**

The QI Maturity Tool - Modified Ontario Version was used to assess the state of QI maturity in 34 PHUs across Ontario. QI maturity was assessed through 23 questions across three dimensions: QI Organizational Culture; QI Capacity and Competency; and QI Perceived Value. QI maturity scores were classified into five stages: Beginning; Emerging; Progressing; Achieving; and Excelling. QI maturity scores were calculated for each of the 34 participating PHUs to determine their stage of QI maturity. Each PHU’s score was then used to determine the provincial average for QI maturity. Participants were also asked to answer three questions related to core CQI organizational structures.

**Results:**

Across the 34 PHUs, 3503 staff participated in the survey. A review of individual PHU scores indicates that Ontario’s PHUs are at varying stages of QI maturity. The average QI maturity score of 4.94 for the 34 participating PHUs places the provincial average in the “Emerging” stage of QI maturity. By QI dimensions, the participating PHUs scored in the “Emerging” stage for QI Organizational Culture (5.09), the “Beginning” stage for QI Competency and Capacity (4.58), and the “Achieving” stage for QI Perceived Value (6.00).

**Conclusion:**

There is an urgent need for Ontario’s PHUs to progress to higher stages of QI maturity. Participants place a high value on QI, but collectively are at less “mature” stages of QI in relation to QI organizational culture and the competency and capacity to engage in QI activities. PHUs should leverage the value that staff place on QI to foster the development of a culture of QI and provide staff with relevant knowledge and skills to engage in QI activities.

## Background

While continuous quality improvement (CQI) has been studied and discussed in Ontario’s public health units (PHUs) for over a decade, understanding of CQI management principles and implementation of quality improvement (QI) practices varies considerably among PHUs across the province. CQI has been defined as “*a management philosophy that focuses on processes and systems rather than the performance of individuals. It uses objective data to analyze and continually improve those processes and address the needs of both internal and external customers. It links data collection, reporting, monitoring and learning and makes them the cornerstones of an ongoing, quality improvement cycle.*” [1] QI can be thought of as the deliberate and defined processes and methods that are used to continuously develop, design, evaluate and change practices and programs to ensure that they are of high quality.

There is a limited body of evidence on QI in public health and a lack of consensus on optimal methods and applications, which creates a gap in the guidance on how to operationalize QI in PHUs [2, 3]. Consequently, this makes it difficult to share information, learn from one another, and develop common standards of practice. Much of the existing evidence base comes from the United States (US) [2]. Research in the US has largely focused on describing efforts to conduct public health QI interventions [4, 5], exploring factors that act as facilitators and barriers to QI efforts and the development of QI culture in PHUs [6–12], and assessing the state of QI and the level of QI maturity in PHUs using the QI Maturity Tool [6, 9–11, 13–18]. While the American literature provides a foundational understanding of QI in public health and helps to inform QI work, extrapolation of findings to Canadian PHUs is limited due to the unique public health landscape in Canada (e.g., funding and organizational structures). Investigation into the level of QI maturity in Canadian PHUs is warranted as a starting point to fill this gap in the evidence base.

The revised OPHS were updated in 2017 and came into effect in 2018. Established by the Ministry of Health for the provision of mandatory health programs and services, the revised standards emphasize the importance of CQI, namely that PHUs across the province “shall ensure a culture of quality and continuous organizational self-improvement” [19]. With this emphasis on CQI, there is a need to understand the level of QI maturity in Ontario’s PHUs.

The CQI Locally Driven Collaborative Project (LDCP) (2015–2021) was established to strengthen CQI in Ontario’s PHUs. This research project brought together relevant stakeholders, including staff from 30 PHUs, academic partners, and experts from Public Health Ontario. The CQI LDCP team worked collaboratively to answer the following research question: “How can systematic CQI be strengthened within Ontario’s PHUs?”. In Phase 1 (2015–2017) of the CQI LDCP, the team conducted a scoping review [20] and assessed the current state of CQI in Ontario’s PHUs. The findings of the latter component of this research project are presented in this paper. The objective of this research was to establish a baseline understanding of QI maturity in Ontario’s PHUs.

## Methods

The CQI LDCP team used the QI Maturity Tool - Modified Ontario Version, to assess the state of QI maturity in 34 PHUs across Ontario. This tool was developed and used in the US, with a specific focus on the public health sector [13, 16, 17], and was subsequently validated in an Ontario PHU. The validation of this survey occurred through a confirmatory factor analysis that was completed with 315 surveys of the original QI tool with a PHU in Ontario. The analysis demonstrated a solid three factor scale with good internal consistency evaluating three distinct aspects of QI (i.e., Culture, Capacity, and Value) and capable of distinguishing differences between groups. This resulted in one of the questions being dropped from the survey as it did not load into any of the factors. Upon review of this question, it was determined that it was reflective of the American public health context and likely not fully understood by Ontario PHU staff. The research team also tested the feasibility of the survey with 15 public health professionals who completed the survey, provided completion time, and engaged in a discussion about the questions and further modifications required to ensure the questions reflected the Ontario PHU context. Three questions were deemed inappropriate for a scale type answer and were rewritten as “yes” and “no” questions.

**Fig. 1:**
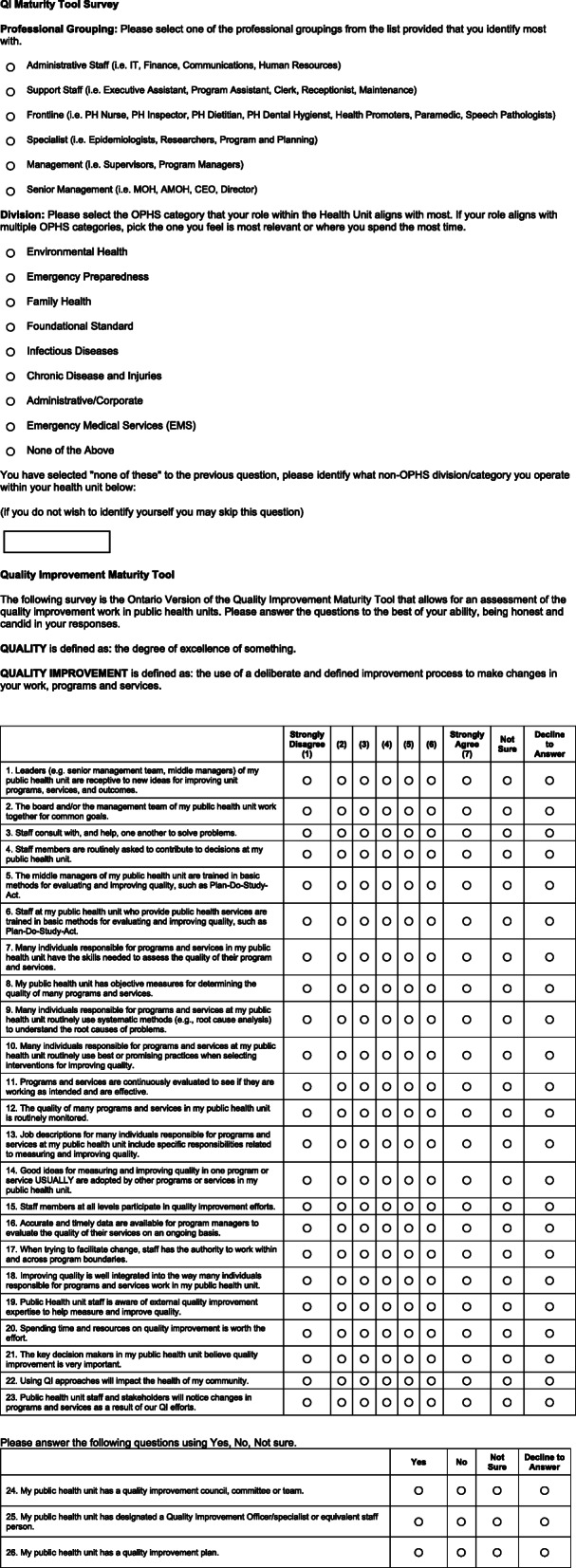
QI Maturity Tool – Modified Ontario Version

The final QI Maturity Tool - Modified Ontario Version consists of 23 questions to assess QI maturity across three dimensions: QI Organizational Culture; QI Capacity and Competency; and QI Perceived Value (Fig. 1). Within the context of the survey, these dimensions are defined as: 1) QI Organizational Culture – The values and norms about QI that pervade throughout the organization relative to how the PHU interacts with staff and stakeholders; 2) QI Capacity and Competency – The skills, functions, and approaches used to assess and improve quality in an organization; and 3) QI Perceived Value – The perceptions of employees that QI is a priority in the organization and supported by leaders while also having the potential to impact services and the community.

The tool is used to calculate QI maturity scores, which correspond to five stages of QI maturity: Beginning (< 4.78); Emerging (4.79–5.12); Progressing (5.13–5.79); Achieving (5.80–6.71); and Excelling (> 6.72). Within the context of the survey, these stages are defined as:

- Beginning – Have not adopted formal QI projects, applied QI methods in a systematic way, or engaged in efforts to build a culture of QI.
- Emerging – Newly adopted QI approaches, albeit with limited capacity. They have a limited QI culture and few, if any, examples of attempts to incorporate QI as a routine part of practice.
- Progressing – Some QI experience and capacity but often lack commitment, have minimal opportunity for QI integration throughout the agency and are less sophisticated in their application and approach.
- Achieving – Fairly high levels of QI practice, a commitment to QI and an eagerness to engage in the type of transformation change described by QI experts.
- Excelling – Achieving high levels of QI sophistication and a pervasive culture of QI.

The survey was launched via FluidSurveys on October 5th, 2016 and closed on November 15th, 2016. Access to the survey was sent via email to all staff at the 34 participating PHUs (*n* = 7515). CQI LDCP members promoted the survey within their respective PHUs. Each question in the survey was answered on a scale of one to seven, with one being “strongly disagree” and seven being “strongly agree”. Participants were also provided the option to select “not sure” for each of the questions. Responses of “not sure” were excluded from the calculations. The average score for each question was calculated for each of the 34 participating PHUs. The average of those scores was then used to determine the stage of QI maturity for each PHU. Individual reports were prepared and distributed for each of the 34 participating PHUs. The scores for each of the 34 participating PHUs were then used to determine the provincial average for QI maturity, consisting of an overall aggregate score as well as aggregate scores for each of the three dimensions. This method was used so that each question and each PHU was weighted equally in the calculation of the provincial average for QI maturity.

Participants were asked to answer three additional questions related to core CQI organizational structures: (1) “My PHU has a QI council, committee, or team”; (2) “My PHU has a designated QI Officer/Specialist or equivalent staff person”; and (3) “My PHU has a QI plan”. Participants were provided the option to select “yes”, “no”, or “not sure” for these questions.

## Results

**Fig. 2:**
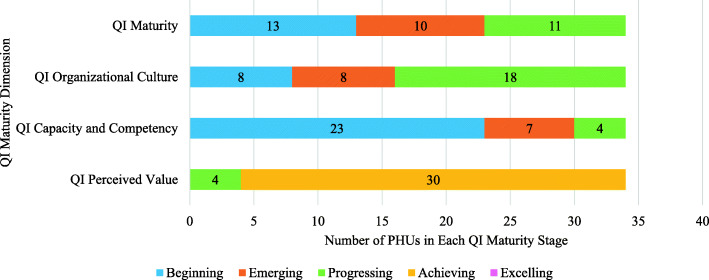
QI Maturity Tool Results for 34 PHUs in Ontario, Canada

Out of 36 PHUs in Ontario at the time of data collection, 34 participated in this survey. Two PHUs declined to participate due to competing priorities (i.e., accreditation process and internal reviews) at the time of the survey. From these 34 PHUs, 3503 staff participated in the survey, resulting in a response rate of 46.6%. The average QI maturity score for all PHUs across the province was 4.94, placing the provincial average in the “Emerging” stage of QI maturity. A review of individual PHU scores indicates that Ontario’s PHUs are at varying stages of QI maturity. Across all 34 participating PHUs, 32% (*n* = 11) scored in the “Progressing” stage; 30% (*n* = 10) scored in the “Emerging” stage; and 38% (*n* = 13) scored in the “Beginning” stage (Fig. 2). No PHU had an average score that would fall within the “Achieving” or “Excelling” stages.

Participants were asked to select one of eight divisions most closely aligned with the division, department, program, or area in which they work. An average QI maturity score was calculated for each division (Table 1). The Administrative/Corporate division (*n* = 379) scored the highest (5.20), placing it in the “Progressing” stage, while the Foundational Standard division (*n* = 212), which is a unit that provides functional decision support, communications, and leadership to the other divisions, scored the lowest (4.30), placing it in the “Beginning” stage.

**Table 1:** QI Maturity Results by Division and Professional Grouping

	Total Participants (n)	QI Maturity		Dimension 1: QI Organizational Culture		Dimension 2: QI Capacity and Competency		Dimension 3: QI Perceived Value	
		QI Maturity Score	QI Maturity Stage	QI Maturity Score	QI Maturity Stage	QI Maturity Score	QI Maturity Stage	QI Maturity Score	QI Maturity Stage
**Division**									
Administrative/Corporate	379	5.20	Progressing	5.30	Progressing	4.88	Emerging	6.15	Achieving
Chronic Disease and Injuries	570	4.86	Emerging	5.04	Emerging	4.46	Beginning	6.07	Achieving
Emergency Medical Services	129	4.78	Beginning	4.76	Beginning	4.63	Beginning	5.43	Progressing
Emergency Preparedness	20	4.95	Emerging	5.10	Emerging	4.67	Beginning	6.20	Achieving
Environmental Health	380	4.88	Emerging	5.07	Emerging	4.59	Beginning	5.71	Progressing
Family Health	946	5.06	Emerging	5.13	Progressing	4.76	Beginning	6.02	Achieving
Foundational Standard	212	4.30	Beginning	4.91	Emerging	3.61	Beginning	6.07	Achieving
Infectious Diseases	739	4.79	Emerging	4.94	Emerging	4.41	Beginning	5.93	Achieving
**Professional Grouping**									
Frontline Staff	2229	4.91	Emerging	5.00	Emerging	4.57	Beginning	5.95	Achieving
Administrative Staff	189	5.01	Emerging	4.83	Emerging	4.76	Beginning	5.86	Achieving
Support Staff	417	5.30	Progressing	5.19	Progressing	5.11	Emerging	5.99	Achieving
Specialist	238	4.38	Beginning	4.84	Emerging	3.79	Beginning	5.90	Achieving
Management	346	4.95	Emerging	5.57	Progressing	4.41	Beginning	6.23	Achieving
Senior Management	168	5.10	Emerging	5.69	Progressing	4.54	Beginning	6.41	Achieving

QI maturity score and QI maturity stage for QI maturity, QI organizational culture, QI capacity and competency, and QI perceived value by division and professional grouping

Participants were also asked to select one of six professional groupings most closely aligned with their role. An average QI maturity score was calculated for each professional grouping (Table 1). Support staff (*n* = 417) scored the highest (5.30), placing this group in the “Progressing” stage, while specialist staff (*n* = 238) scored the lowest (4.38), placing this group in the “Beginning” stage.

### QI organizational culture

The QI Organizational Culture dimension was assessed by examining questions one to four of the survey (Fig. 1). The provincial average for this dimension was 5.09, placing it in the “Emerging” stage of QI maturity. Across all 34 participating PHUs, 52.9% (*n* = 18) scored in the “Progressing” stage; 23.5% (*n* = 8) scored in the “Emerging” stage; and 23.5% (*n* = 8) scored in the “Beginning” stage (Fig. 2). No PHU had an average score that would fall within the “Achieving” or “Excelling” stages. The Administrative/Corporate division (*n* = 379) scored the highest (5.30), placing it in the “Progressing” stage, while the Emergency Medical Services division (*n* = 129) scored the lowest (4.76), placing it in the “Beginning” stage (Table 1). Senior management (*n* = 168) scored the highest (5.69), placing this group in the “Progressing” stage, while administrative staff (*n* = 189) scored the lowest (4.83), placing this group in the “Emerging” stage (Table 1).

### QI capacity and competency

The QI Capacity and Competency dimension was assessed by examining questions five to 19 of the survey (Fig. 1). The provincial average for this dimension was 4.58, placing it in the “Beginning” stage of QI maturity. Across all 34 participating PHUs, 11.8% (*n* = 4) scored in the “Progressing” stage; 20.6% (*n* = 7) scored in the “Emerging” stage; and 67.6% (*n* = 23) scored in the “Beginning” stage (Fig. 2). No PHU had an average score that would fall within the “Achieving” or “Excelling” stages. The Administrative/Corporate division (*n* = 379) scored the highest (4.88), placing it in the “Emerging” stage, while the Foundational Standard division (*n* = 212) scored the lowest (3.61), placing it in the “Beginning” stage (Table 1). Support staff (*n* = 417) scored the highest (5.11), placing this group in the “Emerging” stage, while specialist staff (*n* = 238) scored the lowest (3.79), placing this group in the “Beginning” stage (Table 1).

### QI perceived value

The QI Perceived Value dimension was assessed by examining questions 20 to 23 of the survey (Fig. 1). The provincial average for this dimension was 6.00, placing it in the “Achieving” stage of QI maturity. Across all 34 participating PHUs, 88.2% (*n* = 30) scored in the “Achieving” stage and 11.8% (*n* = 4) scored in the “Progressing” stage (Fig. 2). No PHU had an average score that would fall within the “Excelling” stage. The Emergency Preparedness division (*n* = 20) scored the highest (6.20), placing it in the “Achieving” stage, while the Emergency Medical Services division (*n* = 129) scored the lowest (5.43), placing it in the “Progressing” stage (Table 1). Senior management (*n* = 168) scored the highest (6.41), placing this group in the “Achieving” stage, while administrative staff (*n* = 189) scored the lowest (5.86), also placing this group in the “Achieving” stage (Table 1).

### Additional CQI related questions

Three additional questions were asked related to core organizational structures in place to support CQI efforts in Ontario’s PHUs. For the statement, “My PHU has a QI council, committee, or team”, 48% of participants responded “yes”, 14% responded “no”, and 37% responded “not sure”. For the statement, “My PHU has a designated QI Officer/Specialist or equivalent staff person”, 52% of participants responded “yes”, 11% responded “no”, and 37% responded “not sure”. For the statement, “My PHU has a QI plan”, 42% of participants responded “yes”, 10% responded “no”, and 48% responded “not sure”.

## Discussion

The average QI maturity score of 4.94 for the 34 participating PHUs places the provincial average in the “Emerging” stage of QI maturity. A review of individual PHU scores indicates that PHUs across Ontario are at varying stages of QI maturity, but none are in the “Achieving” or “Excelling” stages, despite many having indicated that they have core CQI organizational structures in place. Research from the US shows that American PHUs are also at varying stages of QI maturity, and that much like Ontario’s PHUs, most are in the “Beginning”, “Emerging”, and “Progressing” stages [13, 15]. However, unlike Ontario’s PHUs, research from the US shows that some American PHUs are in the “Achieving” and “Excelling” stages. This reveals that Ontario’s PHUs are collectively at a lower stage of QI maturity in comparison to their American counterparts. This is not surprising given that American PHUs have been employing QI for a longer period, and are supported with resources and training (e.g., National Association of County and City Health Officials QI Roadmap), as well as accreditation [21–24]. Illustratively, Verma reports that since the Public Health Accreditation Board implemented its voluntary accreditation program in 2011, most local and state health departments in the US have been accredited [23]. Currently, 75% of all health departments in the US (local, state, tribal, territorial, army) are accredited, with another 25% in the process of being accredited. Evidently, there is an urgent need for Ontario’s PHUs to progress to higher stages of QI maturity in order to meet the requirement outlined in the OPHS that PHUs “shall ensure a culture of quality and continuous organizational self-improvement” [19]. This focus on accreditation is interesting as there are no accreditation requirements for PHUs in Ontario, although some do pursue accreditation through various organizations. The influence that voluntary accreditation has on the culture of QI warrants further investigation.

By QI dimensions, the participating PHUs scored in the “Emerging” stage for QI Organizational Culture, the “Beginning” stage for QI Competency and Capacity, and the “Achieving” stage for QI Perceived Value. This demonstrates that participants place a high value on QI (6.00), but collectively are at less “mature” stages of QI in relation to QI organizational culture (5.09) and the competency and capacity to engage in QI activities (4.58). The CQI LDCP scoping review revealed the importance of QI organizational culture and QI competency and capacity as critical elements needed to create, support, and sustain CQI in PHUs [20]. PHUs should leverage the value staff place on QI to foster the development of a culture of QI and provide staff with relevant knowledge and skills to engage in QI activities. Finally, the survey results revealed that divisions and professional groupings are at disparate levels of QI maturity. It is possible that this is partially because certain divisions and professions are more amenable to QI frameworks for their work compared to others. For example, nursing practice in public health requires a continuous review of current practice to meet professional association standards [25]. It is also interesting to note that the Administrative/Corporate division (*n* = 379) scored the highest (5.20), which suggests that those in management positions are much more optimistic, perhaps somewhat unrealistically, about the level of QI maturity compared to those working on the front line. Nevertheless, PHUs should leverage the knowledge and skills of the divisions and professional groupings with the highest QI maturity scores to advance QI maturity within the divisions and professional groupings with the lowest QI maturity scores [16].

While this work can be used to enhance public health professionals’ and researchers’ understanding of the current state of QI maturity in Ontario’s PHUs, it is not without limitations. Firstly, the use of self-reported surveys has inherent flaws that are difficult to control. These include the potential for participant bias (i.e., those with more knowledge and experience in CQI may have filled out the survey), measurement bias due to lack of understanding of the survey questions (e.g., participants might not be clear at which level of the organization they are meant to respond), and social desirability bias (i.e., choosing a higher rating to appear better than the reality). However, strategies were employed to reduce these issues. Participants were informed that the survey results would be anonymous to encourage them to be open and honest in their responses. The survey was sent to all staff at each of the 34 participating PHUs in order to maximize our reach to staff across the PHUs and not just those with knowledge or interest in CQI. Second, regarding the choice of division and professional grouping, it is not possible to state with absolute certainty that participants chose the most appropriate category. As PHUs have different organizational structures, varying job titles, and different professional roles and responsibilities, this presented challenges related to defining categories that would unequivocally apply to all PHUs. The research team used the OPHS standards and core professional groupings to set the categories to ensure the most accurate approach. Third, the response rates by PHU ranged from 22.8 to 86.7%. However, this range of participant responses by PHUs was controlled for by weighting the results of the individual PHUs equally in the calculation of the provincial average. Finally, the three additional questions that were asked about core CQI organizational structures in Ontario’s PHUs were aimed at examining staff awareness and knowledge of core CQI organizational structures in Ontario’s PHUs and may not provide an accurate depiction of what actually exists across the province.

The researchers recognize that there are varying structures and demographics associated with PHUs in Ontario. These include aspects such as population size, number of staff and governance structure (e.g., semi-autonomous, regional boards of health). This was not included in the analysis as it would have allowed for the creation of a profile that would easily identify the PHUs involved. Given that all PHUs must abide by the OPHS and engage in CQI, it was determined that having an analysis combining all the PHUs would allow for a robust understanding of the Ontario context while maintaining confidentiality for the sites.

## Conclusion

To the authors’ knowledge, this is the first study to examine the state of QI maturity in Ontario’s PHUs. It is anticipated that this research will enhance public health professionals’ and researchers’ understanding of the current state of QI maturity in Ontario’s PHUs. The findings of this research informed the work that was done in Phase 2 (2018–2021) of the CQI LDCP focused on a case analysis to support action to enhance QI [26]. In many cases, the individual reports that were prepared and distributed for each of the 34 participating PHUs have motivated them to enact changes to progress to higher stages of QI maturity. As such, future research should assess the progress that has been made to advance the state of QI maturity in Ontario’s PHUs. Further research is warranted, particularly in an Ontario context, to understand what types of structures, practices, and supports are required to enable PHUs to progress to higher stages of QI maturity. With the emphasis on CQI in the revised OPHS, it is more important than ever to strengthen CQI in Ontario’s PHUs.

## Abbreviations

CQI: Continuous quality improvement
LDCP: Locally Driven Collaborative Project
OPHS: Ontario Public Health Standards
PHU: Public health unit
QI: Quality improvement
REB: Research ethics board
US: United States
